# FLT3/CD99 Bispecific Antibody–Based Nanoparticles for Acute Myeloid Leukemia

**DOI:** 10.1158/2767-9764.CRC-24-0096

**Published:** 2024-08-07

**Authors:** Atham Ali, Alvin Phan, Vijaya Vaikari, Mincheol Park, Mateusz Pospiech, Ryan Chu, Yiting Meng, J. Andrew MacKay, Houda Alachkar

**Affiliations:** 1 Department of Clinical Pharmacy, USC School of Pharmacy, University of Southern California, Los Angeles, California.; 2 Department of Pharmacology and Pharmaceutical Sciences, USC School of Pharmacy, University of Southern California, Los Angeles, California.; 3 USC Norris Comprehensive Cancer Center, University of Southern California, Los Angeles, California.; 4 Department of Ophthalmology, USC Roski Eye Institute, USC Keck School of Medicine, University of Southern California, Los Angeles, California.; 5 Department of Biomedical Engineering, USC Viterbi School of Engineering, University of Southern California, Los Angeles, California.

## Abstract

Cluster of differentiation 99 (CD99) is a receptor that is significantly upregulated in acute myeloid leukemia (AML). FMS-like tyrosine kinase 3 internal tandem duplication mutation in AML (FLT3-ITD AML) exhibits even higher levels of CD99 expression. Our group previously employed a novel peptide platform technology called elastin-like polypeptides and fused it with single-chain antibodies capable of binding to FLT3 (FLT3-A192) or CD99 (CD99-A192). Targeting either FLT3 or CD99 using FLT3-A192 or CD99-A192 led to AML cell death and reduced leukemia burden in AML mouse models. Here, we report on the development of a novel Co-Assembled construct that is capable of binding to both CD99 and FLT3 and the antileukemia activity of the bispecific construct in FLT3-ITD AML preclinical models. This dual-targeting Co-Assembled formulation exhibits cytotoxic effects on AML cells (AML cell lines and primary blasts), reduced leukemia burden and prolonged survival in FLT3-ITD AML mouse models. Altogether, this study demonstrates the potential of an innovative therapeutic strategy that targets both FLT3 and CD99 in FLT3-ITD AML.

**Significance:**

This study investigates a dual-targeting strategy in acute myeloid leukemia (AML), focusing on FLT3 and CD99. The approach demonstrates enhanced therapeutic potential, presenting a novel option for AML treatment.

## Introduction

Acute myeloid leukemia (AML) is a hematological malignancy involving uncontrolled proliferation and poor differentiation in myeloid progenitor cells (1). The trajectory in patients with AML has shown a favorable progression in recent years as several new agents have been approved for the treatment of AML. However, the prognosis in these patients is still bleak with a 5-year overall survival rate of ∼30% (2–4). It is critical to develop new treatment strategies, particularly in high-risk patients.

FMS-like tyrosine kinase 3 (FLT3), a gene that encodes a tyrosine kinase receptor, is frequently mutated in AML. The most common FLT3 mutation in AML is an internal tandem duplication (FLT3-ITD) which involves 3 to 400 base pairs located in the juxtamembrane domain of the receptor leading to receptor dimerization that is independent of receptor binding to the FLT3 ligand (FL; refs. 5–7). The resulting auto phosphorylation process leads to the upregulation of proliferation and cell survival pathways such as AKT, MAPK, and STAT5 pathways (8). Patients with *FLT3*-ITD mutations exhibit a higher relapse rate and shorter overall survival. Therefore, FLT3 presents a key therapeutic target in the treatment of AML (9). Several FLT3 inhibitors have been developed and approved for the treatment of patients with AML, such as midostaurin, gilteritinib, and recently quizartinib (10–12). Both midostaurin and quizartinib have shown an improvement in median survival when combined with chemotherapy in newly diagnosed patients with AML (10, 11). Gilteritinib alongside chemotherapy significantly prolonged the survival of relapsed/refractory patients with AML (12). Yet, these FLT3 inhibitors have limited clinical efficacy due to primary and acquired resistance (10, 13–15). Therefore, more effective treatments are needed for patients with *FLT3*-ITD AML.

Cluster of differentiation 99 (CD99), also known as single-chain type-1 glycoprotein, is a transmembrane protein encoded by the *MIC2* gene (16). It plays a role in various biological functions such as cell differentiation, adhesion, protein trafficking, and apoptosis (17–21). CD99 is upregulated in several malignancies such as Ewing sarcoma and leukemias, while exhibiting limited expression in normal tissue (22–24). Our group and others have demonstrated that *CD99* is upregulated in AML and expressed at an even higher level in AML with *FLT3*-ITD mutations (22, 25, 26). Mechanistically, targeting CD99 results in the activation of ERK and SRC signaling pathways, both are key signaling mediators in *FLT3*-WT and *FLT3*-ITD AML (17, 27–29). Previously, we developed two novel antibody-based nano formulations that potently target either CD99 or FLT3 *in vitro* and *in vivo* while exhibiting optimal physiochemical properties and pharmacokinetics (30, 31). These multivalent nanoparticles were composed of a single-chain variable fragment (scFv) fused to an elastin-like polypeptide (ELP). Derived from the human tropoelastin protein, ELPs are genetically engineered biopolymers that can self-assemble into nanoparticles for drug delivery. ELPs consist of repeated pentameric motifs with the amino acid sequence (Val-Pro-Gly-X_aa_-Gly)_*n*_ where the guest residue, X_aa_, specifies any amino acid and *n* represents the number of pentapeptide repeats. When ELPs are heated above their tunable inverse-phase transition temperature (*T*_t_), they phase-separate from bulk solution into a secondary liquid phase called a coacervate. This process is reversible, which allows them to be efficiently purified from cell lysates without chromatography. As ELPs are biodegradable, biocompatible, and exhibit low immunogenicity, they have the potential to be excellent therapeutic agents or drug carriers (32, 33).

To further enhance the therapeutic efficacy of ELP-based nanotechnology in treating AML, this study reports the development of a novel dual-targeting modality that potently targets both CD99 and FLT3 by co-assembling equal amounts of CD99-A192 and FLT3-A192 proteins. This manuscript describes the development of the first of its kind CD99-A192/FLT3-A192 nanoparticles that bind to both CD99 and FLT3 and reports the therapeutic effects of this novel dual-targeting approach in AML preclinical models.

## Materials and Methods

### Cloning and purification of ELPs

In previous studies, we successfully generated a fusion protein consisting of an ELP called A192 and an α-CD99 scFv as well as another ELP fusion protein consisting of A192 and an α-FLT3 scFv (FLT3-A192; refs. 30, 31). The amino acid sequence for the heavy and light variable fragments encoding the α-CD99 scFv and α-FLT3 scFv are presented in Table 1. The acquisition of these amino acid sequences and the cloning methods are described previously (30, 31, 35). To purify the ELPs, ClearColi BL21(DE3) Electrocompetent cells (#60810, Lucigen, WI) were transformed with either the A192, CD99-A192, or FLT3-A192 plasmids. After transformation, colonies were picked and cultured in 200 mL of autoclaved Terrific Broth (TB) with 100 μg/mL of carbenicillin at 37°C for 16 to 18 hours. ClearColi BL21 culture (30 mL) was added to an autoclaved 1 L of TB with 100 μg/mL carbenicillin to culture 6 L of bacteria at 37°C. Once the measured optical density at 600 nm (OD_600_) reached 0.6, 500 μL of 1 mol/L isopropyl β-D-1-thiogalactopyranoside (IPTG) was added to each 1 L flask to bring the final concentration to 500 μmol/L IPTG. IPTG induction was allowed to proceed overnight at room temperature. The next day, the bacteria were lysed through sonication and purification of the ELPs were carried out as described previously (30, 31). This purification process involved several cycles of hot and cold centrifugations, called inverse transition cycling (ITC), which took advantage of the ELPs’ temperature-dependent phase transition.

**Table 1:** Summary of expressed polypeptides

Peptide label	Amino acid sequence^a^	*T*_t_ (°C)^b^	Expected MW (kDa)/monomer	Measured MW (kDa)/monomer^c^	Measured MW (kDa)/particle^d^	*R*_h_ (nm)^e^ Mean ± SD	*R*_g_ (nm)^d^ Mean ± % error	*R*_g_/*R*_h_	Shape^f^
A192	MG(VPGAG)_192_Y	57.7	73.6	73.3	ND	8.2 ± 2.2	ND	ND	ND
CD99-A192	CD99^a^-G(VPGAG)_192_Y	43.7	99.2	99.2	40,000	65.5 ± 5.8	88.0% ± 0.5%	1.34	elongated
FLT3-A192	FLT3^g^-G(VPGAG)_192_Y	44.6	100.1	100.0	9,900	50.4 ± 5.1	45.1% ± 0.7%	0.89	spherical
Co-Assembled	CD99^a^-G(VPGAG)_192_Y + FLT3^g^-G(VPGAG)_192_Y	45.9	99.2; 100.1	99.1; 100.1	27,000	69.7 ± 0.2	71.5% ± 0.5%	1.03	elongated

a α-CD99 scFv: MAEVQLVESGGGLVRPGGSLRLSCAASGFTFSSYAMSWVRQAPGKGLEWVSAISGSGGSTYYADSVKGRFTISRDNSKNTLYLQMNSLRAEDTAVYYCAKSHKRFDYWGQGTLVTVSRGGGGSGGGGSGGGGSSELTQDPAVSVALGQTVRITCQGDSLRSYYASWYQQKPGQAPVLVIYGKNNRPSGIPDRFSGSSSGNTASLTITGAQAEDEADYYCNSSFPRTSSVVFGGGTKLTVLGLVPRGS (30, 34).

b Determined using the maximum first derivative of the OD_350_ for 50 μmol/L ELP in dPBS.

c Determined using largest, double-charged peaks observed by MALDI-TOF MS. Expected MW accounted for the expected N-terminal cleavage of Methionine for A192 and CD99-A192 (35).

d Determined using SEC-MALS.

e Determined using DLS at 37°C, PD, polydispersity.

f The *R*_g_/*R*_h_ ratio of CD99-A192, FLT3-A192, and Co-Assembled is consistent with an elongated nanoparticle.

g α-FLT3 scFv: MEVQLVQSGAEVKKPGASVKVSCKASGYTFTSYYMHWVRQAPGQGLEWMGIINPSGGSTSYAQKFQGRVTMTRDTSTSTVYMELSSLRSEDTAVYYCARGVGAHDAFDIWGQGTTVTVSSGGGGSGGGGSGGGGSDVVMTQSPLSLPVTPGEPASISCRSSQSLLHSNGNNYLDWYLQKPGQSPQLLIYLGSNRASGVPDRFSGSGSDTDFTLQISRVEAEDVGVYYCMQGTHPAISFGQGTRLEIKLVPRGS (31).

### CD99-A192 and FLT3-A192 protein refolding and generation of the dual-targeting, Co-Assembled CD99-A192/FLT3-A192 protein

To induce proper formation of disulfide bonds between cysteine residues of the scFv domains, CD99-A192 and FLT3-A192 were refolded. Each ELP was first denatured in a 1:1 mixture of an equilibration solution (20 mmol/L Tris base, 150 mmol/L NaCl, 8 mol/L urea, 500 mmol/L L-arginine, 10 mmol/L β-mercaptoethanol) and dPBS. Following re-solubilization, the entire solution was transferred to a 10 kDa MWCO dialysis tubing (#68100, Thermo Fisher Scientific, Waltham, MA) and subjected to stepwise dialysis at 4°C. The following buffers were prepared and changed at 24-hour intervals: Buffer 1 (20 mmol/L Tris base, 150 mmol/L NaCl, 3 mol/L urea, 500 mmol/L L-arginine, 2 mmol/L GSH, 0.4 mmol/L GSSG); Buffer 2 (20 mmol/L Tris base, 150 mmol/L NaCl, 1 mol/L urea, 500 mmol/L L-arginine, 2 mmol/L GSH, 0.4 mmol/L GSSG); Buffer 3 (20 mmol/L Tris base, 150 mmol/L NaCl, 0.5 mol/L urea, 250 mmol/L L-arginine); Buffer 4 (20 mmol/L Tris base, 150 mmol/L NaCl, 50 mmol/L L-arginine, pH 8.0), Buffer 5 (1× dPBS; ref. 36). To generate the dual-targeting, Co-Assembled CD99-A192/FLT3-A192 protein (Co-Assembled), a 1:1 mass ratio of CD99-A192 and FLT3-A192 was mixed, denatured, and then refolded in one dialysis tubing using the protocol described above.

### ELP concentration measurements

To measure the concentration of the scFv-ELPs, the protein was denatured and diluted in 6 mol/L guanidine hydrochloride (1:6) to inhibit nanoparticle assembly. Beer-Lambert’s equation was used to calculate protein concentration:

$$C_{\text{ELP}}=\frac{A_{280}-A_{350}}{\varepsilon \;l}$$ (1)

In Equation 1, *A*_280_ is the absorbance at 280 nm, *A*_350_ is the absorbance at 350 nm, *ε* is the molar extinction coefficient, and *l* is the path length (cm). The optical absorbance at 280 and 350 nm was measured with a NanoDrop 2000 spectrophotometer (Thermo Fisher Scientific, Waltham, MA), which has an *l* of 0.1 cm. Using the Expasy ProtParam tool, the *ε* of A192 was estimated to be 1490 mol/L^−1^ cm^−1^, which is small because it contains only one tyrosine residue (37). In contrast, the *ε* of CD99-A192 and FLT3-A192 was calculated to be 43,110 mol/L^−1^ cm^−1^ and 41,620 mol/L^−1^ cm^−1^, respectively (30, 31, 37). As Co-Assembled was produced from a mixture of these fusion scFv-ELPs at a 1:1 mass ratio, the average of the *ε* of CD99-A192 and FLT3-A192 was calculated to obtain an *ε* of 42,365 mol/L^−1^ cm^−1^.

### Conjugation with NHS-rhodamine and NHS-fluorescein

A192 and CD99-A192 were labeled with NHS-rhodamine (#46406, Thermo Fisher Scientific, Waltham, MA), while FLT3-A192 was labeled with NHS-fluorescein (FITC; #46410, Thermo Fisher Scientific, Waltham, MA). The labeling protocol was carried out for each protein to achieve an approximately 40% to 70% labeling efficiency. Briefly, 1 mL of protein was mixed with a 2× molar excess NHS-rhodamine or FITC at 4°C for 1 hour under constant rotation. Unbound, free dye was removed using 10 kDa MWCO dialysis tubing (#68100, Thermo Fisher Scientific, Waltham, MA). After 24-hour dialysis, the absorbance of rhodamine-A192 (Rho-A192) and rhodamine-CD99-A192 (Rho-CD99-A192) at 555 nm (A555) and of Flo-FLT3-A192 at 493 nm (A493) was measured using a NanoDrop 2000 spectrophotometer (Thermo Fisher Scientific, Waltham, MA), which has an l of 0.1 cm. The labeling efficiency was calculated using the equations below, where the *ε* of rhodamine is 80,000 mol/L^−1^ cm^−1^ at 555 nm and *ε* of fluorescein is 70,000 mol/L^−1^ cm^−1^ at 493 nm. To label Co-Assembled (Rho-Co-Assembled) with NHS-rhodamine, FLT3-A192 was first labeled with NHS-rhodamine using the same procedure. After unbound, free dye was removed through dialysis, Rho-FLT3-A192 was mixed with unlabeled CD99-A192 at a 1:1 mass ratio and then refolded in one dialysis tubing using the protocol described above. Assuming that all ELP was recovered during the labeling reaction, the labeling efficiency with each fluorophore was determined using Equations 3–5.

$$C_{\mathrm{rhodamine}}=\frac{A_{555}}{\varepsilon \;l}$$ (2)

$$C_{\mathrm{fluorescein}}=\frac{A_{493}}{\varepsilon \;l}$$ (3)

$$L_{\%}=\frac{C_{\mathrm{fluorophore}}}{C_{\mathrm{ELP}}}100\%$$ (4)

### ELP purity

SDS-PAGE and matrix-assisted laser desorption/ionization–time-of-flight mass spectrometry (MALDI-TOF MS) were performed to determine the purity of the ELPs following ITC. A total of 10 μg of ELP was denatured with 5 μL of β-mercaptoethanol, heated at 95°C for 5 minutes, and loaded onto a 4% to 20% precast SDS-PAGE gel (#4561095, Bio-Rad Laboratories, Hercules, CA). The gel was stained with Bio-Safe Coomassie G-250 Stain (#1610786, Bio-Rad Laboratories, Hercules, CA) and imaged with a ChemiDoc Touch Image System (Bio-Rad Laboratories, Hercules, CA). To quantify the purity of the protein, the entire lane was plotted and the area under each peak was calculated using ImageJ v2.0.0 (National Institutes of Health, Bethesda, MD). The purity of the protein, *P*_%_, expressed as a percentage, was obtained using the following equation:

$$P_{\%}=\left(\frac{A_{\mathrm{peak}}}{A_{\mathrm{total}}}\right)\times 100\%$$ (5)

In Equation 4, the area under the peak of the protein of interest (*A*_peak_) was divided by the sum of the area under all the peaks present in the lane (*A*_total_).

To run MALDI-TOF MS, 2.5 μL of ELP (∼2 mg/mL) was denatured with 2.5 μL of β-mercaptoethanol and then diluted with 5 μL of ultrapure water. The mixture was heated at 95°C for 10 minutes, cooled down to room temperature, and centrifuged at 14,000 rpm. The reduced protein was then mixed with 2,6 dihydroxyacetophenone solution (30 mg/mL in 50% acetonitrile:0.1% formic acid) at a ratio of 1:10 (by volume). 0.8 μL of this solution was spotted on a 384-Big Anchor MALDI target and allowed to dry at room temperature. Crystallized samples were then analyzed using Bruker Rapiflex MALDI-TOF MS (Billerica, MA) equipped with a Smartbeam 3D, 10 kHz, 355 nm Nd:YAG laser. The laser parameters were optimized as follows: scan range = 26 µm; number of shots per sample = 1,000; laser frequency = 5,000 Hz. The mass spectrometer was calibrated for high-mass range using protein A and trypsinogen standards under the linear mode. The data were analyzed using flexAnalysis software (Bruker, Billerica, MA) and plotted using GraphPad Prism (San Diego, CA).

### Transition temperature analysis

The inverse phase transition temperature (*T*_t_) of the ELPs was measured via UV/Vis spectrophotometry. Four different concentrations of the ELPs were prepared using 5-fold serial dilutions (50, 10, 2, and 0.4 μmol/L) in dPBS. The ELPs were heated at a rate of 1°C/minute, starting from 15°C to 85°C and the optical density at 350 nm (OD_350_) was measured by the DU800 UV/Vis spectrophotometer (Beckman Coulter, Brea, CA) at every 0.3°C. At 350 nm, neither fusion scFv-ELPs nor plain ELPs contribute significant absorption. The *T*_t_ was determined as the maximum first derivative of the OD_350_ with respect to temperature.

### Measurements of hydrodynamic radius

Dynamic light scattering (DLS) was used to measure the hydrodynamic radius (*R*_h_) of the ELPs. A total of 25 μmol/L of ELP was prepared in dPBS and filtered with a sterile Acrodisc 0.2 μmol/L filter (#4602, Pall, Port Washington, NY). 60 μL of filtered ELP was added to each well of a 384-well plate in triplicates and 15 μL of mineral oil was added to each well to prevent evaporation of the samples. The hydrodynamic radius was measured at 37°C with a DynaPro Plate Reader II (Wyatt Technology, Santa Barbara, CA).

### Measurement of absolute molar mass and weight of CD99-A192, FLT3-A192, and Co-Assembled nanoparticles

Size-exclusion chromatography–multiangle light scattering (SEC-MALS) was performed to estimate the absolute molar mass of the scFv-ELP nanoparticles. The proteins were diluted to 10 μmol/L in 1 mL of dPBS and were passed through a sterile Acrodisc 0.2 μm filter (#4602, Pall, Port Washington, NY). A Shodex protein KW-803 (8.0 mm ID × 300 mm; Showa Denko America, New York, NY) column was equilibrated with PBS before introducing a bovine serum albumin (BSA) control at 5 mg/mL. The control and scFv-ELPs were analyzed by three detectors for each separated fraction: a SYC-LC1200 UV detector (Agilent Technologies, Santa Clara, CA) at 280 nm, a DAWN HELEOS MALS detector (Wyatt Technology, Santa Barbara, CA), and an Optilab rEX differential refractometer (Wyatt Technology, Santa Barbara, CA). Together, these data were used to determine the absolute molar mass and radius of gyration (*R*_g_) on ASTRA 6.1 software (Wyatt Technology, Santa Barbara, CA). The ratio between *R*_g_ and *R*_h_ was used to infer the morphology of CD99-A192, FLT3-A192, and Co-Assembled nanoparticles (38).

### Negative-staining transmission electron microscopy

To visualize the morphology and size of the scFv-ELP nanoparticles, negative-staining transmission electron microscopy (TEM) was performed. 6 μL of scFv-ELP (0.2 μmol/L) was applied to a carbon-coated 400-mesh Formvar grid (#01754-F, Ted Pella, Inc., Redding, CA). After 3 minutes, the samples were air-dried using filter paper, stained with 4% uranyl acetate for 2 minutes, and then air-dried again for 10 minutes. Images were taken at 120,00× magnification and processed using a JEOL JEM-2100 LaB6 transmission electron microscope (JEOL USA, Inc., Peabody, MA).

### Patient sample studies

Human sample collection was approved by the Institutional Review Board (IRB) of the University of Southern California in accordance with the Helsinki Declaration. Patient samples were obtained from both patients with AML and healthy donors. Samples from patients with AML were obtained from patients at the Norris Comprehensive Cancer Center at the University of Southern California following a written informed consent. Human peripheral blood mononuclear cells (PBMC) were isolated from peripheral blood and used fresh for experiments or stored in freezing media in liquid nitrogen for later use.

### Cell culture

MV4-11, U937, and THP-1 cells were purchased from ATCC. These, as well as MOLM-13 cells cultured in our lab were routinely authenticated at the University of Arizona Genetics Core. MV4-11 and MOLM-13 cells express both FLT3 and CD99 receptors and possess the FLT3-ITD mutation. U937 cells are FLT3 negative but express CD99. THP-1 cells are positive for both FLT3 wild-type (WT) and CD99 expression. All cell lines were cultured in Roswell Park Memorial Institute 1640 (RPMI 1640) medium (cat.no:11875-093 Thermo Fisher, MA, USA) supplemented with 10% fetal bovine serum (FBS; cat.no: 10437-028 Thermo Fisher, MA, USA), 100 U/mL of penicillin and 100 U/mL of streptomycin (cat.no 15240062 Thermo Fisher, MA, USA), and 1× L-glutamine (cat.no 25030-081 Thermo Fisher, MA, USA).

### Generation of midostaurin-resistant MV4-11 cell line

MV4-11 cells were treated every 2 to 4 days with increasing concentrations of midostaurin. Starting at 1 nmol/L and increasing the concentration to 5, 10, 20, 40, 50 nmol/L, and finally 100 nmol/L. Dead cells and debris were removed by washing cells with dPBS and live cells were replated and supplemented with midostaurin. Over the course of 4 months of cells being cultured in the presence of midostaurin, MV4-11 cells exhibited increased resistance to midostaurin.

### Viability assay

All cell viability experiments were performed by incubating cells on ice with either control A192, FLT3-A192 (5 and 20 µmol/L), CD99-A192 (5 and 20 µmol/L), a combination of each fusion protein (2.5 µmol/L of each or 10 µmol/L of each), and FLT3-A192/CD99-A192 Co-Assembled (5 and 20 µmol/L) for 20 to 25 minutes on ice and seeded into either a six-well or 12-well suspension plate. The number of live cells were counted at 72 hours using a trypan blue viability stain (cat.no: 15250061 Thermo Fisher, MA, USA). Cell viability was determined by using the ratio of the number of live cells in treated samples to the number of cells in untreated samples. IC_50_ experiments were also conducted using increasing concentrations (0.01, 0.03, 0.1, 0.3, 1, 3, 10, 30 mol/L). These cells were treated on ice in similar conditions to the viability experiments mentioned previously and seeded into 96-well suspension plates in triplicates for 72 hours. Cell viability was determined using the Cell Counting Kit (WST-8/CCK-8; cat.no: ab228554) and adding 10 μL reagent into each well and incubating for 1 to 4 hours at 37°C per the manufacturer’s protocol and absorbance (450 nm) was measured using the BioTek Synergy H1 Hybrid Multi-Mode Reader (BioTek, VT, USA). The difference in viability was measured and the absorbance of the treated wells was normalized to the untreated wells. The IC_50_ was calculated based on nonlinear regression.

### *In vivo* studies

All animal experiments were conducted under the review and approval of the Institution for Animal Care and Use Committee at the University of Southern California Protocol 20581. Approximately, 1–2 × 10^6^ MV4-11 cells were engrafted via intravenously (IV) through the tail vein into 6 weeks old male and female NOD-scid /Il2rg^−/−^ (NSG) mice from Jackson Laboratory (Bar Harbor, ME). Researchers who were blinded to the treatment groups, administered 250 mg/kg of A192, FLT3-A192, CD99-A192, or Co-Assembled to mice starting day 7 or 10 post leukemia engraftment. Studies were terminated when a mouse regardless of treatment group displayed a phenotype consistent with AML in mice, such as a hunch, decreased movement, or decreased curiosity. Upon termination spleen, bone marrow, and blood were collected, and cells were isolated and stained for huCD45 using an anti-huCD45 antibody (cat.no:25-0459-41, eBioscience, CA, USA). Unbound antibody was thoroughly washed and samples were analyzed using flow cytometry [BD Fortessa X20 Cell Analyzer and processed using FlowJo software (BD, Franklin Lakes, NJ, USA)]. Survival studies involved NSG mice engrafted with 2 × 10^6^–2.5 × 10^6^ MOLM-13 or MV4-11 cells. These mice were treated blindly and euthanization was determined by a second researcher.

### Flow cytometry analyses

Flow cytometry was used to assess the binding of FLT3-A192 and CD99-A192 formulations to their targets on leukemia cells. MV4-11 cells (5 × 10^6^) were incubated with FLT3-A192 and CD99-A192, which were labeled with FITC and NHS-rhodamine, respectively. The Co-Assembled formulation consisted of an unlabeled CD99-A192 and NHS-rhodamine-labeled FLT3-A192. Cells were treated at 5 µmol/L on ice for 20 to 25 minutes. Cells were then washed with PBS three times to remove any unbounded fusion proteins. We then measured binding of FLT3-A192 and CD99-A192 by examining the shift in the mean fluorescence intensity using flow cytometry. Competitive binding experiments were conducted by treating 5 × 10^5^ U937 cells with 20 µmol/L of either FLT3-A192, CD99-A192, or Co-Assembled FLT3/CD99-A192. After a 30-minute cold-binding step on ice, cells were washed and incubated with a CD99 commercial monoclonal antibody (cat.no: 11-0997-42 Thermo Fisher, MA, USA) for 20 minutes on ice. These cells were then washed three times to remove non-binding antibodies and CD99 antibodies that were conjugated to FITC were measured by flow cytometry and analyzed like the previously mentioned binding studies. Similar experiments were also conducted using the FLT3 commercial antibody (cat.no: 17-1357-42 Thermo Fisher, MA, USA) and measuring FLT3 surface level expression post treatment and incubation with CD99-A192 (10 and 30 μmol/L) in MV4-11, MOLM-13, U937, and THP-1 cell lines. Measurements and analysis were also conducted using flow cytometry as described above. The samples used in the cell viability experiments were collected and used for apoptosis assays. Using Annexin V (APC) and propidium iodide (PI; PE) according to the protocol provided by the apoptosis detection kit (cat.no: 88-8007-74 Invitrogen), the percentage of APC and PE positive cells were compared between samples. *In vivo* experiments consisted of engrafting cells and measuring the levels of human CD45 using an anti-HuCD45 antibody (cat.no: 25-0459-41 eBioscience, CA, USA) were analyzed using flow cytometry. All flow cytometry experiments were conducted using the BD Fortessa X20 Cell Analyzer and processed using FlowJo software (BD, Franklin Lakes, NJ, USA).

### Immunoblotting

Immunoblotting was conducted by lysing cells using a protease-phosphatase supplemented lysis buffer (cat.no: 8788, Thermo Fisher, MA, USA; cat.no: A32959, Thermo Fisher, MA, USA). The concentrations of proteins were measured using Bicinchoninic acid protein assay reagent (Pierce, Thermo Fisher, MA, USA). Total cellular lysates (10–20 μg per lane) were separated by SDS-PAGE and transferred overnight onto polyvinylidene fluoride membranes (PVDF; cat.no: GE10600021, Sigma Aldrich). The membranes were then blocked with 5% bovine serum albumin and probed with the following antibodies: STAT5 (cat.no: 25656T, Cell Signaling Technology), SRC (cat.no: 2123S, Cell Signaling Technology), Phospho-STAT5 (cat.no: 9351S, Cell Signaling Technology), Phospho-SRC (cat.no: 6943, Cell Signaling Technology), GAPDH (cat.no: HRP-60004, Thermo Fisher, MA, USA), and Horseradish peroxidase (HRP)-conjugated secondary antibodies (Santa Cruz Biotechnology, TX, USA) were used for antibody detection. Immunodetection was achieved by using the SuperSignal Chemiluminescent Substrate mix (Thermo Fisher, MA, USA) and using the Bio-Rad ChemiDoc Gel Imaging Machine for band detection. Membrane stripping was done using the Restore Western Blot Stripping Buffer (cat.no: 21059, Thermo Fisher, MA, USA). Band detection was quantified with Bio-Rad Image Lab Software and values were generated using the following equation:

$$\mathrm{Band}\;\mathrm{Value}=\frac{\mathrm{Protein}\;\mathrm{Kinase}\;\mathrm{Intensity}}{\mathrm{GAPDH}\;\mathrm{Intensity}}$$ (6)

### RNA preparation and sequencing

MV4-11 cells were treated with 25 μmol/L of FLT3-A192, CD99-A192, or Co-Assembled for 24 hours. Cells were centrifuged at 1,300 rpm for 3 minutes and washed twice with 1 mL of dPBS (cat.no: 14190144, Thermo Fisher, MA, USA). Total RNA was isolated from cell pellet using the RNeasy mini kit with on-column DNase digestion (Qiagen, Cat. #74104) according to the manufacturer’s protocol. RNA concentration and integrity were assessed by Thermo Scientific NanoDrop OneC and Agilent high-sensitivity RNA ScreenTape (Agilent Technologies, Cat. #5067-5579) and measured on the 4200 TapeStation System (Agilent Technologies, Cat. #G2991BA). RNA samples were stored at −80°C until they were shipped to Azenta for library preparation and sequencing. Samples were sent out in two separate runs, each run contained duplicate samples of each treatment group, totaling the samples for each group to *n* = 4. Library preparation included mRNA enrichment, fragmentation and random priming followed by first and second strand cDNA synthesis, end repair, 5′ phosphorylation and dA-tailing. Last step included adaptor ligation and PCR enrichment and sequencing.

### Bioinformatics analysis

Initial bioinformatics analyses were conducted by Azenta. Adaptor sequences and poor-quality nucleotides were trimmed by Trimmomatic v.0.36 followed by mapping to GRCh38 reference genome using STAR aligner v.2.5.2b resulting in BAM files. Annotation was conducted by calculation of unique gene hit counts calculated by Subread package v.1.5.2. As each replicate pair (1,2 and 3,4) came from different batches, batch correction was used on the data. Count data were loaded into R v 4.2.2 and transformed into log2(rawdata + 1). The modcombat function of surrogate variable analysis package v 3.46.0 was used to perform batch correction followed by inversion of log2 transformation. Gene set enrichment analysis (GSEA) v 4.3.2. was used on the normalized RNA sequencing (RNA-seq) gene counts to identify gene sets from the Hallmark database (h.all.v2023.2.Hs.symbols.gmt) and generate normalized enrichment score (NES) plots. Gene expression in RNA extracted from cells treated with A192 served as control and differences between treatments and control were determined and used to generate Pearson’s correlation heatmaps. Gene intensity was used to generate a Venn diagram along with generating pathways. Gene expression in RNA extracted from cells treated with A192 served as control.

### Statistical analysis

The Student *t* test or one-way ANOVA followed by the Tukey multiple comparisons test were used to identify significant differences between two or more than two groups, respectively. Survival studies in mice were analyzed using Kaplan–Meier survival analysis, and the log-rank (Mantel–Cox) test was performed. Mice were randomized after engraftment and monitored by a researcher blinded to the treatment groups. All data presented are shown as mean values with standard deviations (SD). A significance level of *P* < 0.05 was considered statistically significant. For GSEA, a false discovery rate (FDR) below 25% was considered significant.

### Data availability

The data generated in this study are available and will be provided upon reasonable request from the corresponding author.

## Results

### Interplay between CD99 and FLT3 in FLT3-ITD AML

**Figure 1:**
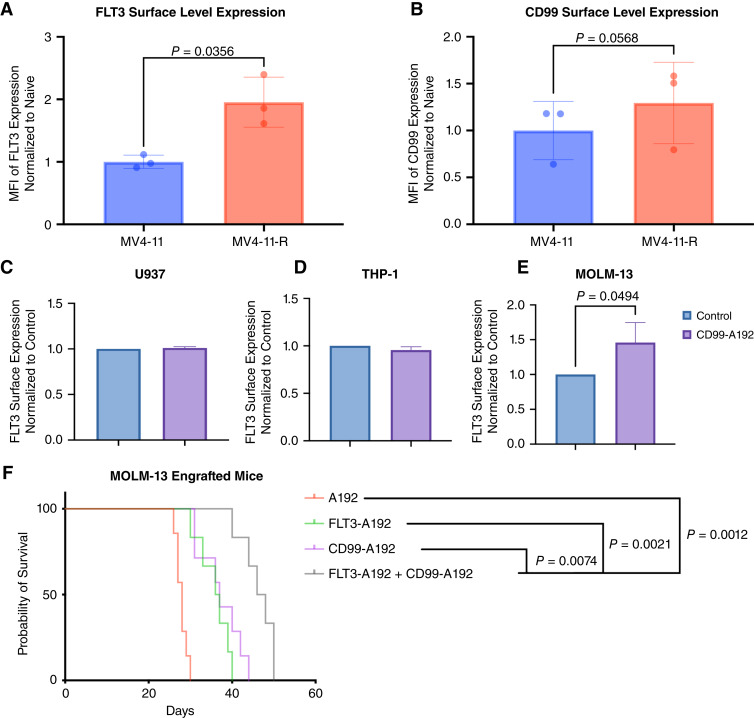
Interplay between CD99 and FLT3 in FLT3-ITD AML. Flow cytometry was used to evaluate the surface level expression of FLT3 and CD99. **A** and **B,** Compared to naive MV4-11 cells, midostaurin-resistant (MV4-11-R) cells have a significant increase of surface expression of FLT3 (*P* = 0.0356), and trend of increase of CD99 (*P* = 0.0568). **C,** To examine the effect of targeting CD99 on FLT3 surface expression, a panel of cell lines were assessed. U937 do not express FLT3 and were included as negative control. **D,** THP-1 cells, although they express the FLT3-WT, did not show enhance FLT3 surface level expression upon treatment with CD99-A192. **E,** In contrast, treatment with CD99-A192 increased FLT3 surface level expression in MOLM-13 cells, which are FLT3-ITD^+^ (*P* = 0.05). **F,** NSG mice were engrafted with 2.5 × 10^6^ MOLM-13 cells, showed a significant increase in survival when treated with both FLT3 and CD99 targeted nanoparticles *P* < 0.007 and *P* < 0.003 of combination vs. CD99-A192, FLT3-A192 alone respectively.

To examine the interplay between CD99 and FLT3, MV4-11 cells were cultured with increasing concentration of midostaurin (10–100 nM) for several months. Resistant MV4-11 cells showed an increase of both FLT3 and CD99 surface expression levels (Fig. 1A and B). Furthermore, MOLM-13 cells (FLT3-ITD cells) treated with 10 μmol/L CD99-A192 showed an increase in FLT3 surface expression levels after 2 hours of treatment (*P* < 0.05, 46% increase). However, this effect was not seen in THP-1 cells, which express FLT3-WT or in U937 cells that lack FLT3 (Fig. 1C–E). Our group previously reported prolonged overall survival of MOLM-13 engrafted mice when treated with CD99-A192. Considering the enhanced surface expression of FLT3 in cells treated with CD99-A192, we speculated that targeting both CD99 and FLT3 is more effective than targeting either receptor. In FLT3-ITD AML xenograft mouse in which 2.5 × 10^6^ MOLM-13 cells were engrafted in NSG mice, the effectiveness of the combination of CD99-A192 plus FLT3-A192 was compared with that of either fusion protein alone. Mice were IV injected via tail vein on day 7, 10, 13, and 16 post-engraftments with 175 mg/kg per mouse of either FLT3-A192 (*N* = 6) or CD99-A192 (*N* = 7) or 87.5 mg/kg of each ELP (*N* = 6), or equal volume of A192 control (*N* = 7). Survival analysis showed that mice treated with the combination of CD99-A192 plus FLT3-A192 survived significantly longer than mice treated with either antibody alone or control A192–treated mice [47 days for the combination vs. 37 and 36.5 days for CD99-A192 or FLT3-A192, respectively, and 28 days for A192 control mice, log-rank (Mantel–Cox) *P* < 0.0001; Fig. 1F].

### Characterization of anti-CD99/anti-FLT3 bispecific scFv-ELP nanoparticles

**Figure 2:**
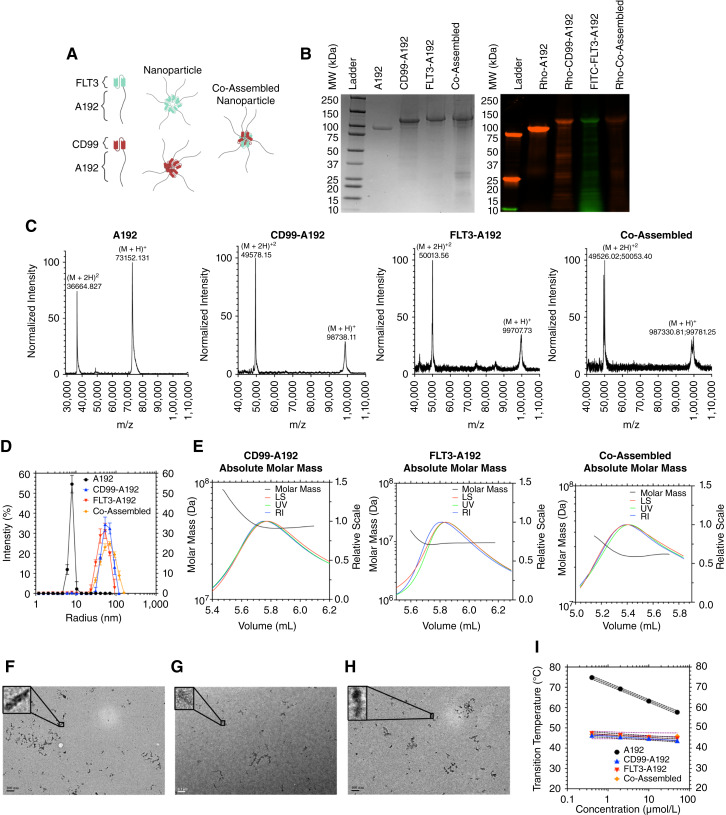
Construction and biophysical characterization of an ELP fusion protein targeting both CD99 and FLT3 receptors. **A,** A single-chain variable fragment targeting either CD99 receptor or FLT3 receptor was genetically fused to the amino-terminus of a high molecular weight ELP, A192. These CD99-A192 and FLT3-A192 nanoparticles were expressed in *E. coli*. Prior to protein refolding, CD99-A192 and FLT3-A192 were co-assembled to generate the dual-targeting CD99-A192/FLT3-A192 nanoparticles. **B,** Following purification, ELPs were obtained with high purity as indicated by an SDS-PAGE gel stained with Coomassie brilliant blue. On the right image, the fluorescent image of the SDS-PAGE gel shows efficient fluorescent labeling the proteins with NHS-rhodamine or FITC. **C,** The high purity of the ELPs was further confirmed by MALDI-TOF MS. The mass spectrum of Co-Assembled shows two sets of distinct m/z peaks corresponding to the presence of both CD99-A192 and FLT3-A192. **D,** The hydrodynamic radius (*R*_h_) of the scFv-ELPs was measured using DLS at 37°C. Compared to CD99-A192 and FLT3-A192, Co-Assembled increased the *R*_h_ of nanoparticle formation. **E,** SEC-MALS was used to measure the absolute molar mass and radius of gyration (*R*_g_) of the scFv-ELP nanoparticles. For all three fusion proteins, the monomers oligomerized to form nanoparticles with an absolute molar mass of 10^7^ to 5 × 10^7^ Da. **F–H,** The morphology of the (**F**) CD99-A192, (**G**) FLT3-A192, and (**H**) Co-Assembled nanoparticles was observed via negative-staining TEM, which showed an elongated, rod-like nanostructures. Scale bar: 0.1 μm. **I,** The transition temperatures (*T*_t_) of the ELPs were measured at 0.4, 2, 10, and 50 μmol/L. Consistent with previous findings, the fusion of scFv to A192 lowers the transition temperature of A192; however, all formulations are expected to remain soluble at physiological temperatures.

Considering the enhanced activity of the combined targeting of CD99 and FLT3, we developed new nanoparticles that are capable of binding both CD99 and FLT3 (Fig. 2A). To generate the dual-targeting CD99-A192/ FLT3-A192 protein (Co-Assembled), CD99-A192 and FLT3-A192 were mixed at a 1:1 mass ratio and then were subjected to protein refolding. The purified ELPs were then characterized for purity and molecular weight using SDS-PAGE and MALDI-TOF MS (Fig. 2B–C; Table 1). A192 appeared as a major band around 75 kDa while the scFv-ELPs appeared as a major band around 100 kDa, which corresponds to the observed and predicted molecular weights determined from MALDI-TOF MS. Additionally, the mass spectrum of Co-Assembled shows two sets of distinct m/z peaks, suggesting the presence of both CD99-A192 and FLT3-A192 that were Co-Assembled. The purity of A192, CD99-A192, FLT3-A192, and Co-Assembled was ∼99%, ∼92%, ∼99%, and ∼59%, respectively. Furthermore, fluorescent imaging of an SDS-PAGE gel with NHS-rhodamine- and FITC-labeled ELPs indicated efficient labeling of the proteins (Fig. 2B).

The scFv-ELPs’ ability to form nanoparticles was confirmed by DLS and SEC-MALS. DLS analysis showed that CD99-A192 and FLT3-A192 formed nanoparticles with a hydrodynamic radius (*R*_h_) of 65.5 ± 5.8 nm (±SD) and 50.4 ± 5.1 nm (±SD) nm, respectively (Fig. 2D; Table 1). Co-assembly of these two nanoworms resulted in larger nanoparticles that have an R_h_ of 69.7 ± 0.2 nm (±SD). On the other hand, A192’s *R*_h_ was 8.2 ± 2.2 nm (±SD), which is representative of the size of a free polymer. Next, the absolute molar mass and radius of gyration (*R*_g_) of the scFv-ELP nanoparticles was determined by SEC-MALS. Monomers of CD99-A192, FLT3-A192, and Co-Assembled were shown to oligomerize into nanoparticles with an absolute molecular weight ranging from 10^7^ to 5 × 10^7^ Da (Fig. 2E; Table 1). After measuring the *R*_g_ of these oligomerized scFv-ELPs, the *R*_g_/*R*_h_ ratio was calculated to predict the shape factor (38). With *R*_g_/*R*_h_ ratios ranging from 1.0 to 1.3, we inferred that CD99-A192 and Co-Assembled exhibit elongated, rod-like nanostructures. In contrast, the *R*_g_/*R*_h_ ratio of FLT3-A192 was calculated to be 0.9, which suggests a spherical shape. While FLT3-A192’s theoretical shape factor differs from the *R*_g_/*R*_h_ ratio of 1.1 reported previously, negative-staining TEM images confirms the elongated morphology of all three constructs. A decrease in the *R*_g_/*R*_h_ ratio may be attributed to batch-to-batch variability (Fig. 2F–H; ref. 31).

To characterize the ELP behavior of the ELPs, the transition temperature (*T*_t_) was obtained by UV–vis spectrophotometry whereby the optical density at 350 nm (OD_350_) was measured over a range of temperatures and ELP concentrations. As shown in Fig. 2I and Table 1, CD99-A192 and FLT3-A192 separate above 44°C and 45°C, respectively (50 μmol/L in dPBS). Co-assembly of both fusion ELPs resulted in a similar phase transition behavior, with a *T*_t_ of 46°C at 50 μmol/L. In contrast, A192 phase separated above 58°C (50 μmol/L in dPBS). Consistent with our previous work, this demonstrates that the attachment of α-CD99 scFv and/or α-FLT3 scFv reduces the phase diagram curve with respect to free A192 (30, 31). For all proteins, the *T*_t_ is sensitive to the ELP concentration; however, the *T*_t_ of the three scFv-ELPs was less sensitive to concentration than A192’s.

### CD99 and FLT3 dual-targeting nanoparticles exhibit binding and specificity in AML cells

**Figure 3:**
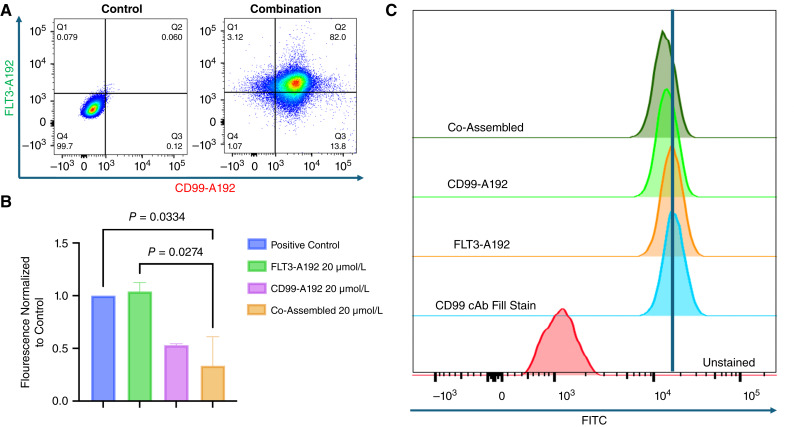
Both antibody fusions exhibit binding and specificity in MV4-11 and U937 cells. **A,** CD99-A192 (Rhodamine) and FLT3-A192 (FITC) bound to MV4-11 AML cells (FLT3-ITD+/CD99^+^). **B** and **C,** In a competitive binding assay using U937 cells (FLT3^−^/CD99^+^), we treated U937 cells with all three of our fusion proteins followed by incubation with a FITC conjugated CD99 commercial antibody (cAb) and measured CD99 cAb FITC signal using flow cytometry. We observed inhibition within both the CD99-A192 and Co-Assembled-treated groups compared with the control group.

The binding of fusion proteins to MV4-11 cell was assessed using flow cytometry. Labeling CD99-A192 with NHS-Rhodamine and FLT3-A192 with FITC, resulted in an increase in the population of events positive for both NHS-rhodamine and FITC (Fig. 3A). Previous studies by our group have illustrated the specificity of each mono-assembled formulation alone. Using the new Co-Assembled formulation, a competitive binding assay showed its specificity for CD99 receptor sites. Using U937 (CD99^+^/FLT3^−^) administered with the Co-Assembled (20 μmol/L) formulation then probing for CD99 receptor sites with a commercial CD99 monoclonal antibody conjugated with FITC showed a decrease in FITC signal of cells treated with the Co-Assembled formulation. When including the mono assembled CD99 formulations (20 μmol/L) a reduction in the shift in comparison to the control was found. Pretreatment with FLT3-A192 (20 µmol/L) did not result in any inhibition (Fig. 3B and C). This further illustrates the specificity of co-assembled nanoparticles.

### Anti-CD99/anti-FLT3 bispecific scFv-ELP nanoparticles exhibit antileukemic activities in AML cell lines and primary blasts

**Figure 4:**
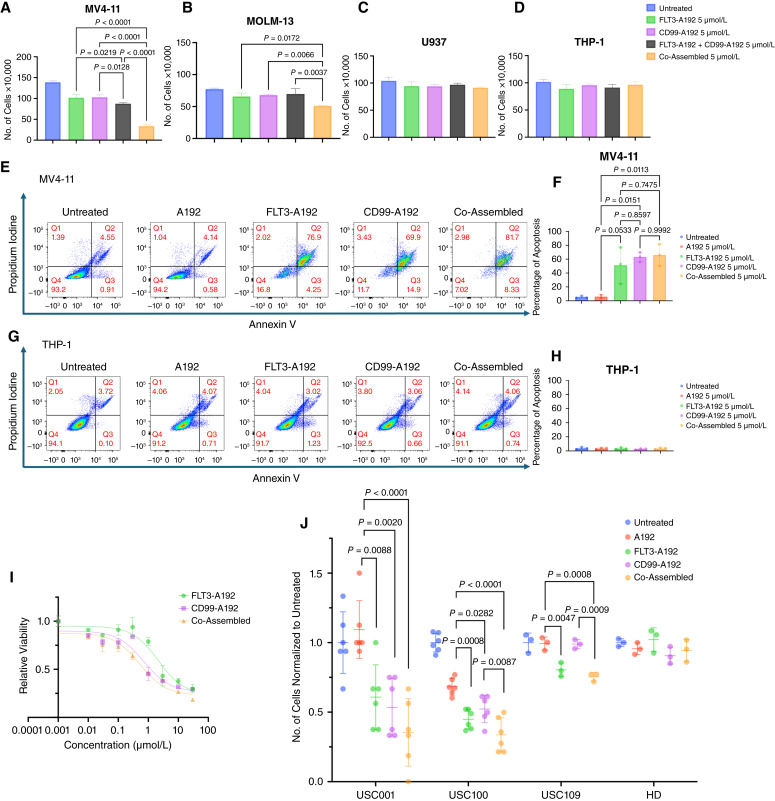
Antibody nanoworms FLT3-A192 and CD99-A192 induce apoptosis and cell death. **A–D,** Trypan blue staining detects a decrease in cell viability across different cell lines FLT3-ITD+/CD99^+^ AML cells (MV4-11, MOLM-13), FLT3−/CD99^+^ cells (U937), and FLT3WT+/CD99^−^ (THP-1). In MV4-11, co-assembling CD99-A192 and FLT3-A192 further reduced viability compared to a combination “mixture” of/or single-targeted nanoworms. **E** and **F,** Greater levels of apoptosis (quadrant Q2) were observed by flow cytometry in MV4-11 cells treated with FLT3-A192, CD99-A192, and Co-Assembled formulations. **G** and **H,** In THP-1 cells levels of apoptosis did not change between the different treatment groups. **I,** The CCK-8 assay was used to quantify the dose-dependent cell viability in MV4-11 cells. Nonlinear curve fitting was used to estimate the IC_50_, which showed that Co-Assembled CD99-A192 and FLT3-A192 (0.2 μmol/L) was more potent than FLT3-A192 (1.7 μmol/L) and CD99-A192 (0.4 μmol/L) by itself. **J,** Effects of FLT3-A192, CD99-A192, and co-assembled in primary patient samples obtained from USC001 (FLT3-ITD+), USC100 (Refractory/Relapse FLT3-ITD+), USC109 (FLT3-ITD + at diagnosis), and healthy donors (HDs). Mean ± SD (*n* = 3).

To assess the dual-targeting approach on FLT3-ITD leukemic cells, the cytotoxicity of the Co-Assembled nanoparticles was tested in a panel of AML cell lines, MV4-11 and MOLM-13 cells which both possess the FLT3-ITD mutation and overexpression of CD99, U937 cells which are absent of FLT3 but express CD99, and THP-1 cells which express the FLT3-WT and CD99. Cells were treated with 5 μmol/L of, FLT3-A192, CD99-A192, or FLT3/CD99 Co-Assembled, or 2.5 μmol/L of each mono-assembled formulation (FLT3-A192 and CD99-A192). Cells were counted using the trypan blue cell viability dye at 72 hours. The number of viable MV4-11 cells was significantly reduced in the treatment groups when compared with the untreated cells. Furthermore, Co-Assembled treated group exhibited significantly higher reduction of cell viability compared with other treatment groups (Co-Assembled vs. FLT3-A192, *P* < 0.0001, 49% decrease; Co-Assembled vs. CD99-A192, *P* < 0.0001, 50% decrease; Co-Assembled vs. FLT3-A192 + CD99-A192, *P* < 0.0001, 38% decrease; Fig. 4A). The number of MOLM-13 viable cells was significantly decreased in the Co-Assembled group vs. the different treatment groups (Co-Assembled vs. FLT3-A192, *P* = 0.02, 19% decrease; Co-Assembled vs. CD99-A192, *P* = 0.006, 22% decrease; Co-Assembled vs. FLT3-A192 + CD99-A192, *P* = 0.004, 24% decrease; Fig. 4B). There was no difference in both U937 cells and THP-1 cells between the untreated group and the treatment groups (Fig. 4C and D).

To assess if the fusion proteins induced antibody dependent cellular cytotoxicity through apoptosis, MV4-11 and THP-1 cells were stained with Annexin V and PI to measure apoptosis 72 hours after treatment. Apoptosis was slightly greater in cells treated with 5 μmol/L of Co-Assembled in comparison to other treatment groups (FLT3-A192 vs. A192, 45% increase; CD99-A192 vs. A192 57% increase; Co-Assembled vs. A192 60% increase), no effect was observed in THP-1 cells (Fig. 4E–H) or in U937 cells (Supplementary Fig. S1) when cells were treated with 5 μmol/L of either fusion protein. Using a cell viability kit, IC_50_ values of different treatment groups (FLT3-A192, CD99-A192, and Co-Assembled) in MV4-11 cell lines were found to be 1.7 μmol/L (95% CI, 1.0–2.8), 0.4 μmol/L (95% CI, 0.2–0.7), and 0.2 μmol/L (95% CI, 0.1–0.4) respectively (Fig. 4I). These findings suggest the Co-Assembled formulation has a similar IC_50_ to that of CD99-A192, with the Co-Assembled formulation having a sub-micromolar IC_50_.

The cytotoxic effects of the formulations on primary samples from AML patients with FLT3-ITD mutations both refractory/relapse FLT3-ITD+, and FLT3-ITD+ at diagnosis were examined. After isolating the PBMC from these samples, the expression levels of both FLT3 and CD99 were assessed by flow cytometry (Supplementary Fig. S2). Once confirmed, these cells were treated with FLT3-A192, CD99-A192, and FLT3/CD99 Co-Assembled at 20 μmol/L. A significant decrease in the number of live cells at 72 hours was observed in all three treatment groups of a newly diagnosed FLT3-ITD AML sample (USC001) compared with A192-treated group (FLT3-A192 vs. A192, *P* = 0.009, 45% decrease; CD99-A192 vs. A192, *P* = 0.002, 51% decrease; Co-Assembled vs. A192, *P* < 0.0001, 67% decrease; Fig. 4J). Similarly, in USC100, obtained from a patient with refractory/relapsed FLT3-ITD AML, this sample showed a significant decrease in the number of cells at 72 hours in all three treatment groups compared with A192-treated group (FLT3-A192 vs. A192, *P* = 0.0008, 34% decrease; CD99-A192 vs. A192, *P* = 0.03, 24% decrease; Co-Assembled vs. A192, *P* < 0.0001, 49% decrease) as well as between CD99-A192 and Co-Assembled (Co-Assembled vs. CD99-A192, *P* < 0.009, 36% decrease; Fig. 4J). Cells from USC109, obtained from a patient with FLT3-ITD AML at diagnosis exhibited a significant decrease in cell viability at 72 hours in FLT3-A192 and Co-Assembled compared with A192 but not in CD99-A192 (FLT3-A192 vs. A192, *P* = 0.005, 19% decrease; Co-Assembled vs. A192, *P* = 0.0008, 24% decrease; Co-Assembled vs. CD99-A192, *P* = 0.0009, 24% decrease; Fig. 4J). Within healthy donor (HD) samples, no changes were found between the different treatment groups (Fig. 4J).

### Anti-CD99/anti-FLT3 bispecific scFv-ELP nanoparticles exhibit antileukemic activities *in vivo*

**Figure 5:**
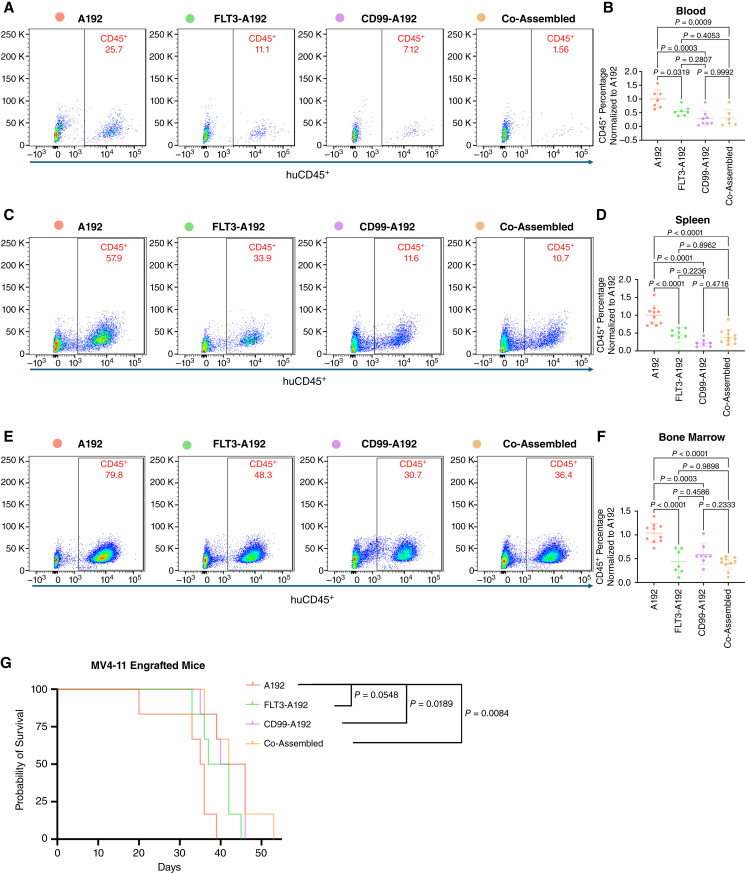
Treatment with fusion protein formulations significantly reduced leukemia burden in NSG mice. **A–C,** 2 × 10^6^ MV4-11 cells were engrafted IV into NSG mice. Mice were then treated with either A192, FLT3-A192, CD99-A192, or Co-Assembled at a dose of 250 mg/kg every other day beginning at either day 7 or day 10, ending between day 21 to 28. **A** and **B,** Peripheral blood samples, (**C** and **D**) spleen samples, and (**E** and **F**) bone marrow samples show a lower percentage of huCD45^+^ cells between A192 and all three treatment groups. **G,** We engrafted 2 × 10^6^ MV4-11 cells into NSG mice and treated these mice at day 8, 10, 12, 14, 16, 18, 20, and 22 post engraftment, Kaplan–Meier survival analysis shows that mice treated with Co-Assembled survived longer than mice receiving FLT3-A192 alone. Mean ± SD (*n* > 5).

To quantify the *in vivo* effects with these fusion proteins, the MV4-11 NSG-mouse model was employed. NSG-mice were engrafted via tail vein injection with 2 × 10^6^ MV4-11 cells and treated with A192, FLT3-A192, CD99-A192, or Co-Assembled at 250 mg/kg administered via tail vein injection. The mice were then euthanized, and their tissue samples were collected such as the blood, spleen, and bone marrow for analysis. Flow cytometry was used to measure the percentage of cells stained with Anti-HuCD45 antibodies. A significant reduction in the percentage of blood huCD45 cells was found in all three treatment groups compared with the vehicle A192 group (FLT3-A192 vs. A192, *P* = 0.03, 44% decrease; CD99-A192 vs. A192, *P* = 0.0003, 70% decrease; Co-Assembled vs. A192, *P* = 0.0009, 69% decrease; Fig. 5A and B). In spleens, there was also a significant reduction in huCD45 percentage between A192 and all three treatment groups (FLT3-A192 vs. A192, *P* < 0.0001, 55% decrease; CD99-A192 vs. A192, *P* < 0.0001, 77% decrease; Co-Assembled vs. A192, *P* < 0.0001, 62% decrease; Fig. 5C and D). Consistently, the percentage of huCD45 leukemia cells were significantly reduced in bone marrows collected from the fusion protein–treated mice compared with the control A192 groups (FLT3-A192 vs. A192, *P* < 0.0001, 58% decrease; CD99-A192 vs. A192, *P* = 0.0003, 41% decrease; Co-Assembled vs. A192, *P* < 0.0001, 60% decrease; Fig. 5E and F). Survival analyses were conducted in the MV4-11 xenograft mouse model. NSG mice (*n* = 6 per group) were treated with 250 mg/kg of either A192, FLT3-A192, or CD99-A192 and their survival was followed. A significant increase in survival was shown for mice treated with CD99-A192 and Co-Assembled formulations when compared with A192 (CD99-A192 vs. A192, *P* = 0.02, median survival 35.5 vs. 43; Co-Assembled vs. A192, *P* = 0.01, median survival 35.5 vs. 44; Fig. 5G). There was no statistical difference between Co-Assembled versus CD99-A192 (*P* = 0.6) or Co-Assembled versus FLT3-A192 (*P* = 0.2).

### Anti-CD99/anti-FLT3 bispecific scFv-ELP nanoparticles exhibit antileukemic effects in midostaurin-resistant cells

**Figure 6:**
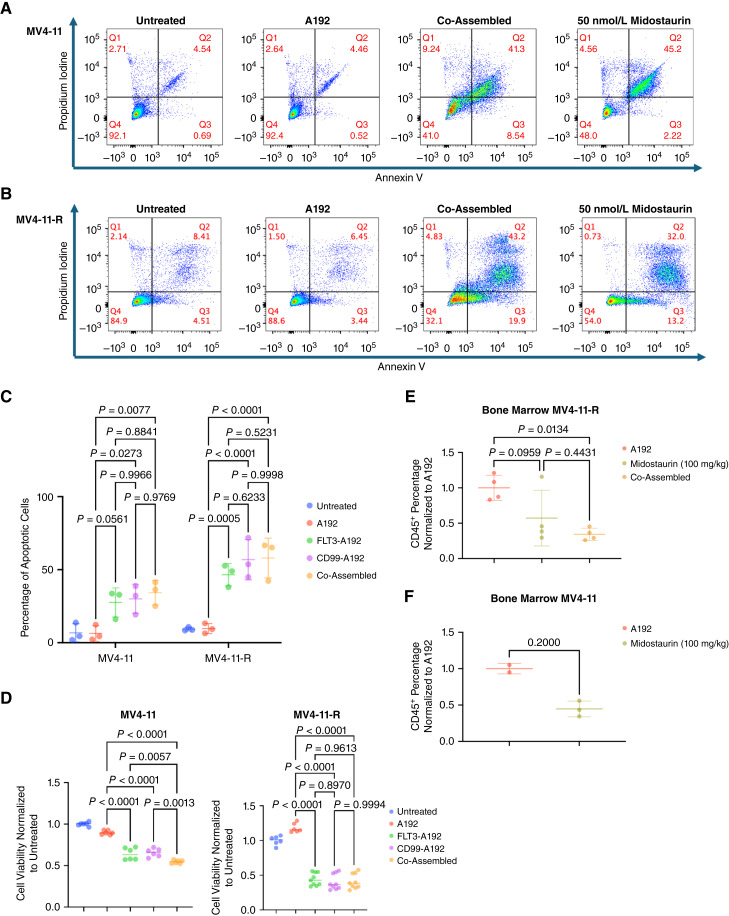
Midostaurin-resistant MV4-11 cells show sensitivity to CD99/FLT3 antibody-based nanoparticles. MV4-11 cells were selected with resistance to increasing concentrations of midostaurin during several months of culture and evaluated by flow cytometry. **A–C,** Apoptosis experiments illustrate how the live-cell (Q4) percentage for midostaurin-resistant MV4-11 cells is decreased when compared to naïve cells, eluding at their maintained and increased sensitivity to the different treatment formulations. **D,** The antibody-based nanoparticles were evaluated against both naïve and resistant cell lines, which show that the nanoparticles maintain their effect in resistant MV4-11 cells, illustrated by the reduction of cell viability. **E** and **F,** Engraftment of 10^6^ MV4-11-R cells in NSG mice following the same treatment regiments as previous MV4-11 *in vivo* experiments show a significant decrease in the percentage of huCD45 cells between A192 and the Co-Assembled construct. Additionally, naïve MV4-11 cells were engrafted as a control to illustrate the effect of midostaurin.

Midostaurin is frequently used to treat AML, but patients can develop resistance to this tyrosine kinase inhibitor, rendering the treatment ineffective. This resistance was evident in the case of USC100, which led to an adverse outcome. Midostaurin-resistant MV4-11 cells were employed to investigate the efficacy of the developed fusion proteins in overcoming midostaurin resistance. Treatment of both MV4-11 and MV4-11-R cells with the fusion proteins resulted in a significant increase of apoptotic events when compared with A192. Within MV4-11-R, we observed an increase in apoptosis in all three treatment groups compared with A192-treated cells, (Co-Assembled vs. A192, *P* < 0.0001, 49% increase; CD99-A192 vs. A192, *P* < 0.0001, 47% increase; FLT3-A192 vs. A192, *P* = 0.0005, 37% increase; Fig. 6A–C). No significant changes were observed in MV4-11 cells between Co-Assembled and either FLT3-A192 or CD99-A192 (Co-Assembled vs. FLT3-A192, *P* = 0.88; Co-Assembled vs. CD99-A192, *P* = 0.98; Fig. 6A–C). Similarly, no significant changes were observed in MV4-11-R cells between Co-Assembled and either FLT3-A192 or CD99-A192 (Co-Assembled vs. FLT3-A192, *P* = 0.52; Co-Assembled vs. CD99-A192, *P* > 0.99; Fig. 6A–C). A drastic decrease was also shown in cell viability of both MV4-11 (FLT3-A192 vs. A192, *P* < 0.0001, 26% decrease; CD99-A192 vs. A192, *P* < 0.0001, 24% decrease; Co-Assembled vs A192, *P* < 0.0001, 35% decrease) and MV4-11-R cells (FLT3-A192 vs. A192, *P* < 0.0001, 74% decrease; CD99-A192 vs. A192, *P* < 0.0001, 78% decrease; Co-Assembled vs. A192, *P* < 0.0001, 77% decrease; Fig. 6D). Additionally, NSG mice were engrafted with 1 × 10^6^ MV4-11-R cells. In the midostaurin-treated group, mice received a dosage of 100 mg/kg midostaurin daily from day 7 to day 11. Mice in the A192 and Co-Assembled groups were administered a dose of 250 mg/kg every other day, beginning on day 7 and concluding on day 17. Upon termination at day 21, a significant decrease in the percentage of huCD45 was observed in Co-Assembled-treated mice compared with A192-treated mice (*P* = 0.01, 65% decrease; Fig. 6E). The effect of midostaurin on naïve MV4-11 cells was also demonstrated *in vivo* (Fig. 6F).

### The mechanism of a dual-targeting approach

**Figure 7:**
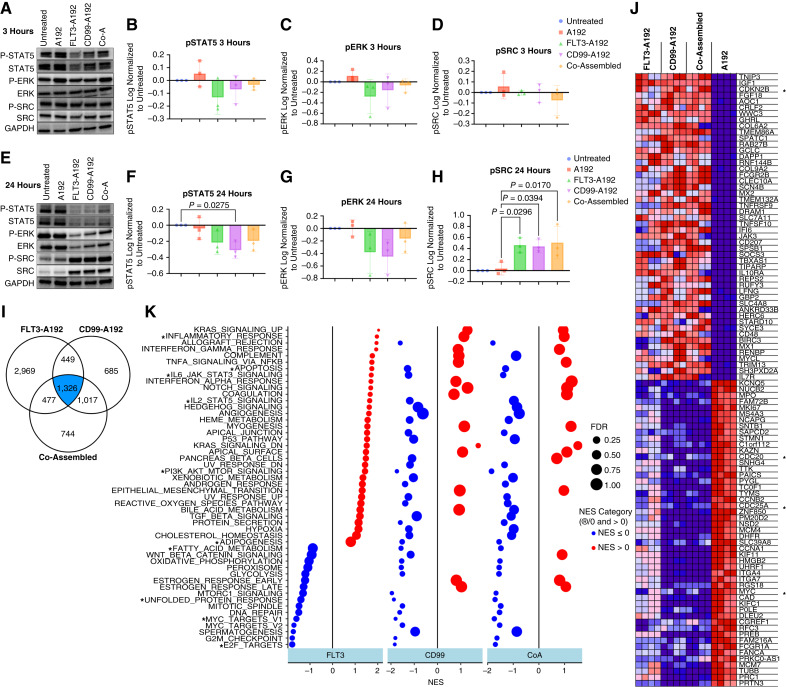
Signaling pathways and genes affected by the treatment with CD99/FLT3 fusion proteins. Following incubation of MV4-11 with treatments or controls, cell lysates were assessed using western blot against common signaling pathways associated with AML. **A–D,** 3 hours timepoints did not show drastic changes in SRC, ERK, and STAT5 pathways. **E–H,** Treatment with targeted fusion proteins reduced phosphorylation of kinases associated with AML. For example, an increase of P-SRC at 24 hours is consistent with CD99 inhibition. Image analysis was used to quantify and compare relative activation of pSTAT5, pSRC, and pERK. **I,** RNA-seq analysis was performed on MV4-11 cells. A Venn diagram shows the number of differentially expressed genes for cells treated with FLT3-A192, CD99-A192, or the Co-Assembled formulation. **J,** Differential RNA expression was analyzed using GSEA. All three treatments cluster for genes associated with cancer growth and progression. **K,** Treatment with FLT3-A192, CD99-A192, and Co-Assembled result in different pathways being enriched and downregulated; FDR < 25%.

To determine the underlying molecular mechanisms that govern FLT3-ITD+ AML, downstream signaling kinases associated with FLT3 and CD99 AML were explored. MV4-11 cells were treated with 25 μmol/L of A192, FLT3-A192, CD99-A192, or Co-Assembled formulations. Cells were collected and lysed at 3 and 24 hours. Signaling proteins associated with FLT3 receptor such as STAT5 and the RAS/MAPK kinase (MEK)/extracellular signal-regulated kinase (ERK) pathways and downstream signaling proteins associated with CD99 such as the SFK family kinase protein SRC, were assessed by western blot at 3 hours (Fig. 7A–D). A decrease of phospho-STAT5 in FLT3-A192-, CD99-A192-, and Co-Assembled-treated groups was observed at 24 hours post treatment (CD99-A192 vs. Untreated, *P* < 0.0381, 50% decrease; Fig. 7E and F). There was also a decrease of phospho-ERK at 24 hours in FLT3-A192-, CD99-A192-, and Co-Assembled-treated groups (Fig. 7G). However, an increase of phospho-SRC at 24 hours in FLT3-A192-, CD99-A192-, and Co-Assembled-treated groups was observed (Co-Assembled vs. A192, *P* < 0.0170, 13-fold increase; CD99-A192 vs. A192, *P* < 0.0394, 11-fold increase; FLT3-A192 vs. A192, *P* < 0.0296, 12-fold increase; Fig. 7H). No differences were observed between the A192 group and the untreated group. Utilizing refractory AML samples from patient, USC100 cells treated with these formulations for 18 hours were similarly evaluated using immunoblotting. These results revealed a decrease of phospho-STAT5 in both FLT3-A192 and Co-Assembled groups (Supplementary Fig. S3). There was an increase of phospho-ERK throughout all treatment groups and a decrease of phospho-SRC in FLT3-A192 and Co-Assembled groups (Supplementary Fig. S3). To better understand the impact fusion proteins targeting FLT3 and/or CD99 have on the RNA level, we conducted RNA-seq analysis on treated MV4-11 cells. These studies revealed both similarities and differences in the expression of genes among the treatment groups (Fig. 7I). Interestingly, 744 unique genes were differentially expressed by the Co-Assembled formulation that were not obtained by either treatment alone. Among the most downregulated genes in all three treatment groups were genes involved in cell cycle progression such as *CDC25A* and *CDC20* and cell proliferation such *MYC* (Fig. 7J). Consistently, genes involved in cell cycle regulation such as *CDKN2B* were found to be upregulated in the treatment groups. Validations of these results were done by qPCR and reported in Supplementary Fig. S4. Treatments with FLT3-A192, CD99-A192, and Co-Assembled all resulted in a significant downregulation of pathways associated with DNA repair, E2F targets, G2M checkpoint, MYC, and unfolded protein response (FDR < 0.25; Fig. 7K). Targeting MV4-11 cells with FLT3-A192 resulted in a significant NES of pathways associated with IL6/JAK/STAT3 signaling, PI3K/AKT/MTOR signaling, inflammation, apoptosis, STAT5-, and P53-related pathways (FDR < 0.25; Fig. 7K). While, CD99-A192 treatment resulted in a significant downregulation of pathways associated with adipogenesis, fatty acid metabolism, and PI3K/AKT/MTOR signaling (FDR < 0.25; Fig. 7K). The Co-Assembled-treated cells also resulted in a significant downregulation of adipogenesis, fatty acid metabolism, IL6/JAK/STAT3, and PI3K/AKT/MTOR signaling pathways (FDR < 0.25; Fig. 7K).

## Discussion

This study introduces a novel dual-targeting fusion protein, effectively targeting both CD99 and FLT3, to enhance treatment efficacy. Both FLT3 and CD99 are promising therapeutic targets in FLT3-ITD+ AML (10, 13, 22, 39). Prior research indicates frequent overexpression and mutation of FLT3 in AML, particularly the ITD mutation. This mutation is linked to adverse prognosis, drug resistance, and relapse (5, 9, 40, 41). Although FDA-approved FLT3-targeting therapies like midostaurin show promise in AML treatment, achieving complete remission remains a challenge, illustrating the demand for innovative treatments (10, 42, 43). CD99 has also been found to be upregulated in various cancers such as AML and Ewing sarcoma (22, 24, 44). CD99 is notably overexpressed in FLT3-ITD+ AML and can modulate the expression of FLT3 on the cell surface when cells are treated with anti-CD99-targeting monoclonal antibodies (26). The concurrent elevation of FLT3 and CD99 in cell lines resistant to midostaurin underscores the potential therapeutic value of simultaneously targeting both receptors. Our research, along with other studies, demonstrates that targeting AML cells through FLT3 or CD99 induces cell death, establishing both receptors as viable therapeutic targets (22, 45). Despite this, treatment strategies simultaneously targeting both receptors have not been explored until now. We hypothesized that targeting both receptors simultaneously with a unique platform peptide technology, elastin-like polypeptides (ELP), would have a stronger antileukemic effect than targeting either receptor alone. Using ELPs fused with a single-chain antibody for FLT3 and CD99, we successfully created a Co-Assembled bispecific nanoparticle, offering fusion protein advantages such as improved pharmacokinetics, biodegradability, and low immunogenicity (46). ELPs phase separate at tunable temperatures, facilitating purification of antibody-based fusion proteins through ITC, eliminating the need for chromatography. A192, CD99-A192, FLT3-A192, and Co-Assembled show purity levels of approximately 99%, 92%, 99%, and 59%, with high yields averaging 68, 94, and 48 mg/L, respectively. ITC prevents inclusion body entrapment, a common issue in single-chain antibody nanoparticle purification. These formulations exhibit stability at 37°C, displaying elongated rod-like nanoworm shape in microscopy. Pharmacokinetic profiles of these formulations have been conducted and show excellent results when compared to the free scFv (30, 31). The terminal half-life of CD99-A192 is 15.8 hours with a mean residence time (MRT) of 21.3 hours, while FLT3-A192 has a terminal half-life of 14.7 hours and an MRT of 18.7 hours (30, 31). The longer half-life can be attributed to the size increase when these fusion proteins form nanoparticles, helping to avoid glomerular filtration, a critical weakness of scFvs (47).

Targeting FLT3 or CD99 induces apoptosis in FLT3-ITD+ AML *in vitro* and reduces leukemia burden *in vivo*. As CD99 is also expressed on normal tissues including mature T cells and monocytes (48), while FLT3 is also expressed on normal hematopoietic stem cells (49), dual-targeting approach is speculated to have higher on-target antileukemia effect and less off-target effects in healthy tissues. The dual-targeting approach demonstrated specificity, effectively achieving therapeutic effects with lower concentrations of FLT3-A192 and CD99-A192. Co-targeting FLT3 and CD99 showed enhanced therapeutic impact compared with single receptor targeting, particularly in FLT3-ITD mutation-positive leukemic cell lines. No significant effects were observed in healthy donor samples, emphasizing the nontoxic nature and specificity of our nanoparticles for FLT3-ITD leukemic cells. The Co-Assembled formulation exhibited improved stability, pharmacokinetics, and lower IC_50_ than mono-assembled formulations, indicating potential for targeted therapy in AML. Our treatment strategy showed positive responses in refractory AML primary samples treated with FLT3-A192, CD99-A192, and Co-Assembled formulations, highlighting the potential of our antibody-based fusion proteins. Additionally, USC109 non-responsiveness to CD99-A192 was subverted with the Co-Assembled formulation, demonstrating the benefit of a dual-targeting treatment design. Exploring different ratios of FLT3-A192 or CD99-A192 in Co-Assembled formulations may enhance therapeutic effects, but uneven ratios due to refolding variations should be considered.

Similar effects were also observed in NSG AML xenograft mouse models, effectively demonstrating the impact of our FLT3 and CD99 targeting fusion proteins *in vivo*. Mice treated with our Co-Assembled formulation exhibited a slightly more favorable effect. Survival studies confirmed these outcomes, with the Co-Assembled formulation marginally surpassing the individual targeting formulations. Mechanistically, the treatment of FLT3-ITD+ MOLM-13 cells with CD99-A192 resulted in an increase in FLT3 surface level expression. Furthermore, MV4-11 cells, which had developed resistance to midostaurin, displayed an increase in FLT3 and CD99 surface level expression. Building upon this mechanistic insight, FLT3-ITD+ MV4-11 cells that had developed resistance to midostaurin exhibited higher sensitivity to fusion proteins compared with MV4-11 naïve cells.

The mechanistic analyses revealed shared pathways being affected by targeting either CD99 or FLT3. Signaling proteins linked to cell growth (pERK, pSTAT5) decreased within 24 hours post-treatment in all MV4-11 groups. Simultaneously, pSRC increased after 24 hours across all groups, aligning with prior findings on CD99 targeting affecting SRC (22, 25). GSEA also showed similar pathways being affected by targeting either CD99 or FLT3 in MV4-11 cells. Genes linked to cell cycle and proliferation, such as *CDC25A* and *CDC20* were differentially expressed in association with the inhibition of cell growth observed *in vitro* (50, 51). Despite a similarity in their effects, FLT3-A192, CD99-A192, and Co-Assembled treatments resulted in distinct genes being differentially expressed. Treatment with FLT3-A192 enriched pathways such as inflammation and apoptosis, along with P53, which correlates with the observed *in vitro* phenotype. MV4-11 cells treated with CD99-A192 or the Co-Assembled show a notable reduction in PI3K/AKT/MTOR related pathways.

Due to its novelty, studies that implement refolding two ELP-based fusion proteins are limited, studies to examine and address these shortcomings such as refolding ratios and refolding retention are ongoing. Targeting both FLT3 and CD99 yielded a similar, if not greater, effect compared to using either single-targeting fusion protein alone. This outcome was accomplished despite refolding half of each single-targeting formulation to generate the Co-Assembled formulation, demonstrating the specificity and efficacy of a dual-targeting treatment strategy. This emphasizes the potential to minimize adverse effects and selectively target leukemic cells due to the fact that less of each nanoparticle are used, however further studies should be conducted to confirm this possibility. In summary, this study compared the mechanistic and efficacy of single- versus dual-targeting treatments for AML. *In vitro* testing of AML cell lines and AML primary samples shows the evidence for better efficiency with two targets as shown in Fig. 4A, B, and J. RNA-seq analysis also shows the clear differences in downstream effects that these constructs have as well. Furthermore, repurposing these nanoparticles and shifting to a cargo delivery design, utilizing small-molecule inhibitors for both FLT3 and CD99 may also yield promising results. This would allow for the design of micelles consisting of ELP’s that can select for either FLT3 or CD99 and a core containing small molecule inhibitors targeting FLT3 and CD99 expressing cells (46). Presently, the landscape of AML treatment is effective with therapies designed for FLT3-ITD AML. However, with the emergence of resistance and limited efficacy associated with FLT3 inhibitors, it becomes imperative to introduce novel treatment strategies that can propel the field of AML treatment and maintenance forward.

## Conclusion

In summary, our study unveils the development and preclinical evaluation of an innovative dual-targeting approach for treating FLT3-ITD+ AML. Leveraging ELPs, we have created a Co-Assembled formulation with the unique ability to target both FLT3 and CD99, the first of its kind. This unique formulation not only enhances pharmacokinetic parameters, stability, and production yield but also demonstrates the effectiveness of exploiting the co-occurrence of CD99 alongside FLT3-ITD mutations in AML. *In vitro* experiments have successfully showcased the therapeutic benefits of simultaneously targeting both FLT3 and CD99 receptors. Our findings indicate that this formulation exhibits prolonged survival in mouse models and holds promise in *in vivo* efficacy studies. The fusion proteins exhibit antileukemic effects in blasts obtained from refractory AML patient, further establishing the clinical significance of our discoveries. These results emphasize the pivotal roles of both FLT3 and CD99 in the context of relapsed or refractory AML, positioning them as promising targets for further evaluation. Our study introduces a novel class of refolded Co-Assembled fusion proteins with potential applications in different disease models and indications.

## Supplementary Material

Figure S1 Figure S1 – Flow Panels of Annexin and PI staining of U937 Cells following treatment with FLT3-A192, CD99-A192 and Co-Assembled CD99-A192 and FLT3-A192.

Figure S2 Figure S2 – FLT3 and CD99 surface level expression in primary blasts of sample USC001 and USC100.

Figure S3 Figure S3 – Western Blot data from USC100 following treatment with FLT3-A192, CD99-A192 and Co-Assembled CD99-A192 and FLT3-A192.

Figure S4 Figure S4 – qPCR analysis results for CDC25A, E2F1, CDKN2B, CDC20, and MYC in MV4-11 cells treated with with FLT3-A192, CD99-A192 and Co-Assembled CD99-A192 and FLT3-A192.
