# Supplementary figures and images for: FLT3/CD99 Bispecific Antibody–Based Nanoparticles for Acute Myeloid Leukemia
Source: Cancer Res Commun. 2024 Aug 7;4(8):1946–62. doi: 10.1158/2767-9764.CRC-24-0096 (PMC11305399; doi:10.1158/2767-9764.CRC-24-0096)

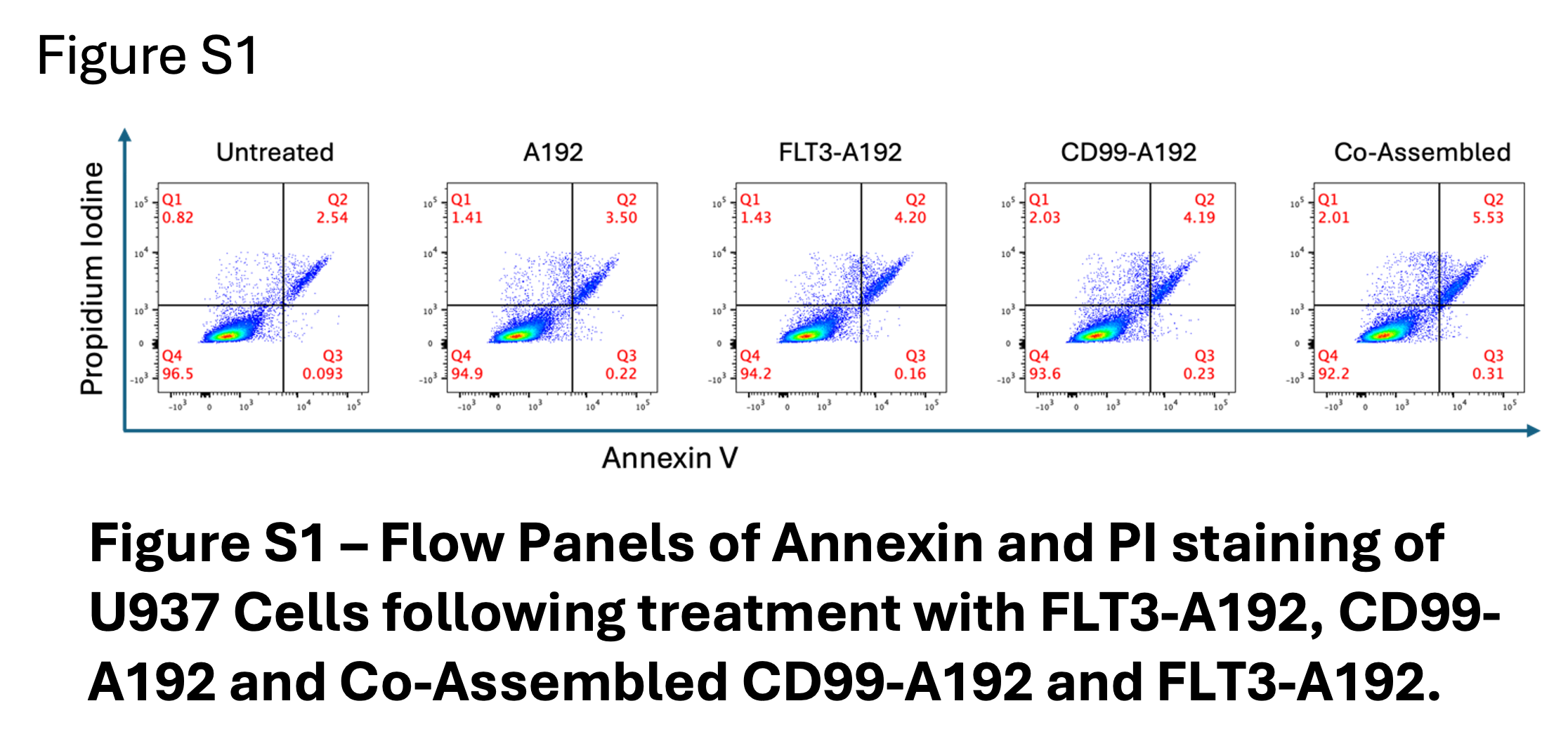

Supplement: Figure S1 — – Flow Panels of Annexin and PI staining of U937 Cells following treatment with FLT3-A192, CD99-A192 and Co-Assembled CD99-A192 and FLT3-A192. [file crc-24-0096_figure_s1_suppsf1.png]

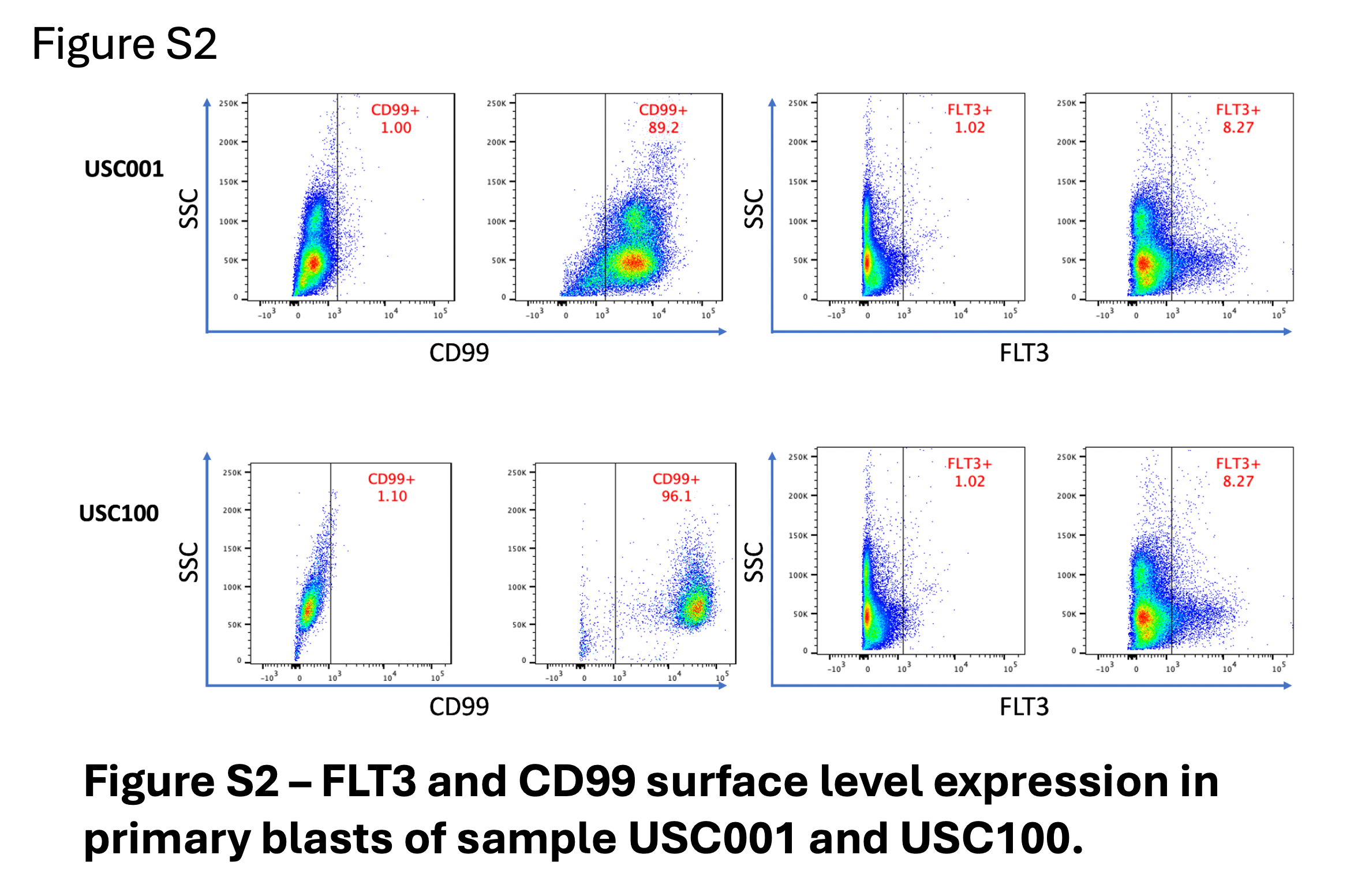

Supplement: Figure S2 — – FLT3 and CD99 surface level expression in primary blasts of sample USC001 and USC100. [file crc-24-0096_figure_s2_suppsf2.png]

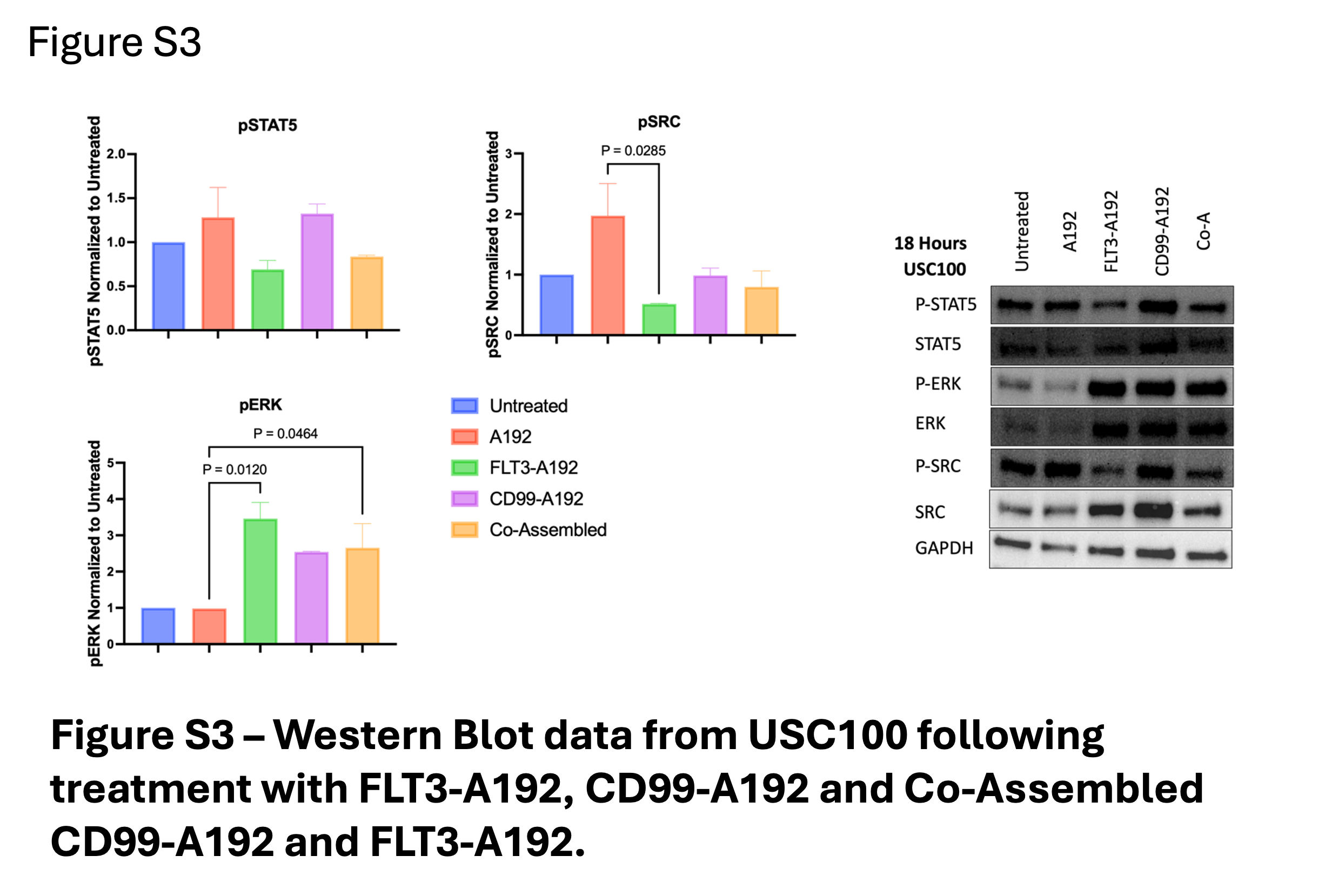

Supplement: Figure S3 — – Western Blot data from USC100 following treatment with FLT3-A192, CD99-A192 and Co-Assembled CD99-A192 and FLT3-A192. [file crc-24-0096_figure_s3_suppsf3.png]

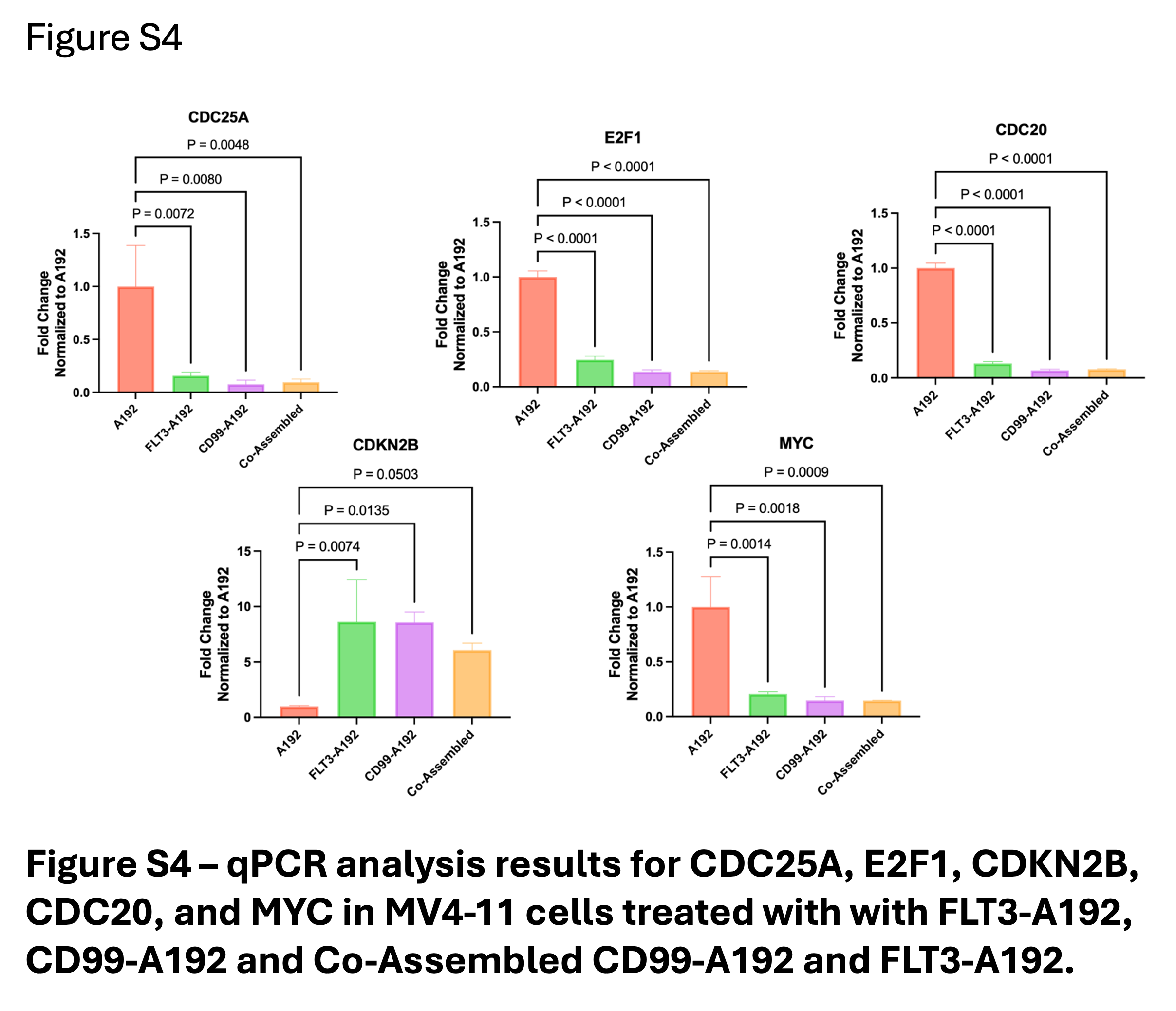

Supplement: Figure S4 — – qPCR analysis results for CDC25A, E2F1, CDKN2B, CDC20, and MYC in MV4-11 cells treated with with FLT3-A192, CD99-A192 and Co-Assembled CD99-A192 and FLT3-A192. [file crc-24-0096_figure_s4_suppsf4.png]
